# An innovative visual acuity chart for urgent and primary care settings: validation of the Runge near vision card

**DOI:** 10.1038/s41433-019-0372-8

**Published:** 2019-02-21

**Authors:** Matthew D. Cooke, Patricia A. Winter, Kaitlin C. McKenney, Krissa L. Packard, Vesper Williams, Eleanor A. Dorsey, Aniko Szabo, Alexis Visotcky, Clinton C. Warren, William J. Wirostko, David V. Weinberg, Judy E. Kim, Dennis P. Han

**Affiliations:** 1 Mayo Clinic Health System, Eau Claire, WI USA; 2 Department of Ophthalmology and Visual Sciences, Medical College of Wisconsin, Milwaukee, WI USA; 3 Institute for Health and Society, Medical College of Wisconsin, Milwaukee, WI USA

**Keywords:** Vision disorders, Physical examination, Eye manifestations, Medical research

## Abstract

**Objective:**

We evaluated the Runge card, a near-vision eye chart designed for ease of use, by testing agreement in visual acuity results between it and the Early Treatment Diabetic Retinopathy Study (ETDRS) visual acuity chart. As a clinical reference point, we compared the Runge card and an electronic Snellen chart with respect to agreement with ETDRS results.

**Methods:**

Participants consisted of adult eye clinic patient volunteers who underwent a protocol refraction, followed by testing with a Runge card, ETDRS chart, and Snellen chart. Mean logMAR visual acuities were calculated for each method. Agreement levels among the tests were assessed for the group overall and for subjects with good (ETDRS logMAR < 0.6; better than 20/80 Snellen equivalent) and poor (logMAR ≥ 0.6) acuity.

**Results:**

One hundred and thirty-eight participants completed testing. The mean ( ± standard deviation) logMAR visual acuities (Snellen equivalent) with Runge, ETDRS, and Snellen, respectively, were 0.66 ± 0.50 (20/91, *n* = 138), 0.59 ± 0.51 (20/78, *n* = 138), and 0.67 ± 0.62 (20/94, *n* = 137). Runge testing agreed similarly with ETDRS and Snellen testing, with CCC 0.92 between Runge and ETDRS, and 0.87 between Runge and Snellen (*p* = 0.14). Runge agreed better with ETDRS than Snellen agreed with ETDRS in participants with poor acuity (CCC = 0.79 vs. 0.63, respectively, *p* = 0.001) but not in those with good acuity (CCC = 0.70 vs. 0.87, respectively, *p* = 0.005).

**Conclusion:**

Visual acuity measurements with the Runge near card agreed with measurements from the ETDRS to approximately the same degree as did the Snellen chart, suggesting potential utility of the Runge near card, particularly given its user-friendly characteristics and ease of use.

## Introduction

We evaluated the Runge Near Vision Card (Runge card, Good-Lite, Elgin, Illinois), a near-vision eye chart with an innovative design intended to deal with some of the drawbacks of most of the currently used near visual acuity tests. These drawbacks include nonstandard progression of letter size and chart lines containing letters of varying number and difficulty level [1]. In addition, patients with poor fixation or cognitive impairment may perform poorly with current charts, as most require that the patient locate the beginning of each line of letters even as the letters become smaller and more challenging to find. Such difficulty could create testing artifact from variable efforts of either the examiner or examinee. The Runge card reduces the need for frequent line changes and begins each line with relatively large letters, potentially making line changes easier. It may be particularly suited to low vision, primary care, and urgent care settings where ease of administration may be important, and where there may be great variation in patient cognition and personnel applying the test. The Runge card also provides the opportunity to test each eye on a different set of letters if so desired.

**Fig. 1:**
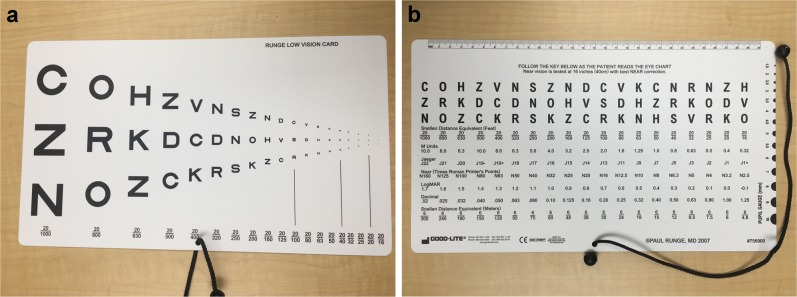
A photograph of the front **a** and back **b** of a Runge near card. The back of the card is viewed by the examiner to score testing. Each sequence of letters is read by the patient left-to right as far as possible before proceeding to read the next sequence. For clinical use, a cord of specific length is provided with the card and affixed to it to facilitate a 16-inch working distance. Note that in **b**, the back of the near card incorrectly lists the M Units for the first three letters as 10.0, 8.0, and 6.3. The M Units for the first three letters are actually 20.0, 16.0, and 12.5. The other M Unit values are listed accurately. (Good-Lite, Elgin, Illinois)

The Runge card uses Sloan letters of similar legibility like those utilized by ETDRS charts, a known standard for clinical research [2, 3]. It features three sequences of letters, with each letter across a sequence corresponding to a progressively higher level of visual acuity (Fig. 1a, b). The letters become progressively smaller from left-to right, with the smallest letter on the right corresponding to a visual acuity of 20/16, (logarithm of minimum angle of resolution [logMAR] −0.1). A patient reads letters across each sequence left-to-right, simplifying testing instructions, and reducing to only twice the need for refixation at the beginning of the line. Like ETDRS, the Runge card uses an equal number of letters per acuity level (three for the Runge card rather than five for the ETDRS, owing to space constraints). In addition, each chart utilizes 10 Sloan letters of similar legibility (C, D, H, K, N, O, R, S, V, Z) [4]. Finally, like the ETDRS chart, owing to its logarithmic progression and equal number of letters per line, the Runge card test can be scored based on number of letters correctly read, and its comparison with the ETDRS chart in the same cohort of subjects could be potentially useful in validating the card. To provide a clinically useful frame of reference we also evaluated how a “Snellen” chart might perform against the ETDRS chart. Such an evaluation can provide only a rough perspective, as “Snellen” charts, vary greatly in appearance and design among manufacturers. It is also known that the uneven progression of acuity lines on many Snellen type charts affects agreement with the ETDRS chart, especially at poor levels of acuity [5, 6]. For purposes of this study, we considered a performance approximately equivalent to a Snellen chart by the Runge card to be an indicator of its potential clinical utility.

## Materials and methods

After obtaining Institutional Review Board approval, a prospective study was performed November 2015 to January 2017. All research was conducted following the Tenets of the Declaration of Helsinki. Participants recruited from clinics of multiple providers met the following inclusion criteria: at least 18 years old; able to speak, read, and understand English; able to give written informed consent; and able to understand and follow protocols for visual acuity testing. We excluded potential participants with right eye acuity of logMAR 1.7 (20/1000) or worse (unable to read any letters on an ETDRS chart one meter from subject).

After written informed consent was obtained, visual acuity testing was performed in a single session by the same examiner with the same lighting conditions. A right eye standard protocol manifest refraction was performed, utilizing ETDRS Chart R (Precision Vision, Woodstock, Illinois). Right eye visual acuity testing was then performed with an ETDRS chart, Snellen chart, and Runge card, with the left eye covered. For ETDRS and Snellen testing, the best correction was placed in a trial frame in front of the right eye. For Runge testing, + 2.25 power was added to adjust for a change in working distance from four meters (refraction at four meters, + 0.25 relative to infinity (1/4 meters)) to 16 inches (0.4 meter distance, + 2.50 relative to infinity (10/4 meters), so difference of + 2.25 was added). The order of acuity testing was determined randomly for each participant.

For ETDRS testing, ETDRS Chart 1 was placed four meters from the participant. Testing was performed as described by Kaiser [5], except if a participant could not read at least three letters on the top line, the chart was placed one meter from the subject, with + 0.75 power added to adjust for the working distance. If a participant could not then read at least one letter on the top row, the participant was excluded from the study. Each correctly read letter was recorded on a scoring sheet, with the number of letters read used to determine the logMAR acuity. For four meter testing, the letters read multiplied by 0.02 was subtracted from 1.1 to determine logMAR acuity.

For Snellen testing, an electronic Snellen chart (M&S Technologies Smart System 20/20, Niles, Illinois) was projected six meters from the participant. Testing started with the top line and continued until a participant incorrectly read half or more letters on a line or read all letters on the chart. If a participant could not read the top letter (20/800 acuity), a letter “E” (size of letter on 20/200 line) was held five feet in front of the participant and moved forward at one-foot intervals until the participant identified the direction of the letter. Acuity was scored as the line of smallest letters at which at least half of letters were read, plus or minus letters on the next or previous line. For example, if a participant read the 20/30 line and one letter on the 20/25 line, the acuity was 20/30 + 1. If the letter “E” was read at four feet, an acuity of 4/200 (20/1000) was used. These scores were converted to logMAR by taking log (base ten) of the Snellen fraction reciprocal. The value of additional letters was calculated by the difference in logMAR between the unmodified line and line with additional letters.

For Runge testing, the card was placed 16 inches in front of the participant. The illuminance of the Runge near card was 500 lux, approximating the brightness of the ETDRS chart as measured by a photometer. Participants read each sequence, with testing continued until a participant read all letters or missed or failed to attempt three consecutive letters. Each correctly read letter was recorded on a scoring sheet, with the number of letters read used to determine logMAR acuity. The letters read multiplied by 1/30 was subtracted from 1.8 to determine logMAR acuity.

The agreement between visual acuity measurements was displayed with Bland–Altman plots and quantified with limits of agreement with standard deviation. Lin’s concordance correlation coefficients (CCC) were used to quantify agreement between visual acuity measurements [7].

Subgroup analyses for participants with “good” (ETDRS logMAR < 0.6; better than 20/80 Snellen equivalent) and “poor” visual acuity (ETDRS logMAR ≥ 0.6) were conducted. These acuity definitions were prespecified before beginning the study. This acuity threshold was selected because it is about halfway between logMAR 0.3 (20/40), a common cutoff for unrestricted driving privileges, and logMAR 1.0 (20/200), the cutoff for legal blindness in the United States, allowing for a detailed analysis around these regulatory cutoffs. The bias between subgroups was compared using Welch’s *t* test, the standard deviation of the difference using the *F* test, and the concordance correlation using *z*-test on Fisher’s z transformed values.

## Results

Initially, 144 participants were evaluated. Six (4.2%) read no letters on an ETDRS chart one meter from the right eye and were excluded. Thus, 138 participants were included, including 67 (48.5%) with good (ETDRS logMAR < 0.6) and 71 with poor acuity (ETDRS logMAR ≥ 0.6). One participant completed testing with Runge and ETDRS only, before the protocol was amended to add Snellen testing.

The median age of participants was 72 years (interquartile ranges [IQRs] 57, 84.5), with significantly older age in participants with poor (median 83 years; IQRs 65, 89) versus good acuity (median 63 years; IQRs 47, 74; *p* < 0.001). Eighty-three (60.1%) were female, including 42 (62.7%) with good and 41 (57.7%) with poor acuity. Thirty-three (23.9%) had no right eye pathology, 39 (28.3%) had exudative age-related macular degeneration (AMD), 17 (12.3%) non-exudative AMD, 8 (5.8%) proliferative diabetic retinopathy, 7 (5.1%) diabetic macular edema, 6 (4.3%) central retinal vein occlusion, 6 (4.3%) retinal detachment, 4 (2.9%) cataract, 3 (2.2%) Stargardt’s Disease, and 15 (10.9%) had various other ocular conditions.

The mean ( ± standard deviation; SD) logMAR visual acuities [Snellen equivalent] with Runge, ETDRS, and Snellen, respectively, were 0.66 ± 0.50 [20/91, *n* = 138], 0.59 ± 0.51 [20/78, *n* = 138], and 0.67 ± 0.62 [20/94, *n* = 137].

**Fig. 2:**
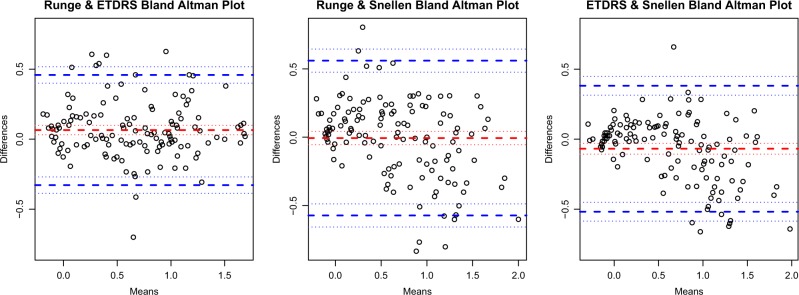
A Bland–Altman plot displaying the differences for each participant in logarithm of minimum angle of resolution (logMAR) visual acuity scores between a Runge near card and an Early Treatment Diabetic Retinopathy Study (ETDRS) distance chart (left plot), between a Runge card and a Snellen distance chart (center), and between ETDRS and Snellen distance charts (right). For each plot, the *x* axis displays the mean logMAR acuity for the two methods being compared. The *y* axis displays the difference in logMAR acuity between the methods compared. Values above zero on the *y* axis represent greater logMAR values for the Runge card than the method being compared in the left and center plots and for the ETDRS chart than the Snellen chart for the right plot. The red-dashed line represents the mean difference in logMAR acuity between the methods being tested, and the bold blue dashed lines represent the limits of agreement, i.e., 1.96 standard deviations from the mean difference in logMAR acuity, approximating an interval that would include 95% of future observations based on estimated mean and variance

Figure 2 shows Bland–Altman plots evaluating differences in visual acuity (bias) between Runge and ETDRS, between Runge and Snellen, and between ETDRS and Snellen testing. The mean (± SD) difference for the Runge-ETDRS comparison was 0.07 ± 0.20 logMAR (positive value indicates higher mean logMAR value (worse acuity) with first listed test, in this case Runge); for Runge–Snellen, −0.01 ± 0.29 logMAR (negative value indicating lower mean logMAR value (better acuity) with first listed test); and for ETDRS–Snellen, −0.07 ± 0.23 (ETDRS with better mean acuity). For the group overall, the Runge card appeared to correlate with the ETDRS at least as well as the Snellen. Lin’s CCC for Runge-ETDRS measurements was 0.92 (95% confidence intervals (CI) 0.89, 0.94); for Runge–Snellen measurements, 0.87 (CI 0.83, 0.91); and for ETDRS–Snellen measurements, 0.91 (CI 0.88, 0.94). There were no significant differences in CCCs among these comparisons.

Subanalyses were performed on data obtained from participants with good vision and from those with poor vision. In those with good vision, the mean (± SD) difference for the Runge-ETDRS comparison was 0.11 ± 0.19 logMAR (Runge with mean acuity about one line worse than ETDRS); for Runge–Snellen, 0.11 ± 0.23 (Runge with mean acuity about one line worse than Snellen); and for ETDRS–Snellen, 0.00 ± 0.14 (mean acuity practically the same between ETDRS and Snellen). Lin’s CCC for Runge-ETDRS measurements was 0.70 (CI 0.58, 0.81); for Runge–Snellen measurements, 0.64 (CI 0.51, 0.78); and for ETDRS–Snellen measurements, 0.87 (CI 0.82, 0.93). The CCC for ETDRS–Snellen measurements was significantly greater than for Runge-ETDRS (*p* = 0.005) and Runge–Snellen (*p* = 0.001) measurements, the latter two of which did not significantly differ from each other (p = 0.66).

In participants with poor vision, the mean ( ± SD) difference for the Runge-ETDRS comparison was 0.02 ± 0.20 logMAR (mean acuity practically the same); for Runge–Snellen, −0.12 ± 0.29 logMAR (Runge with mean acuity about one line better than Snellen); and for ETDRS–Snellen, −0.13 ± 0.27 (ETDRS with mean acuity about one line better than Snellen). In participants with poor vision, the Runge card showed greater correlation with ETDRS than did the Snellen. Lin’s CCC for Runge-ETDRS measurements was 0.79 (CI 0.70, 0.88); for Runge–Snellen measurements, 0.65 (CI 0.53, 0.78); and for ETDRS–Snellen measurements, 0.63 (CI 0.50, 0.77). The CCC for Runge-ETDRS measurements was significantly greater than for ETDRS–Snellen measurements (*p* = 0.001). The CCCs did not differ significantly between the remaining comparisons.

Table 1 shows the numerical values of the limits of agreement for each of the comparisons (i.e., 1.96 standard deviations from the mean difference in logMAR acuity), approximating an interval that would include 95% of future observations based on estimated mean and variance. The 95% estimated limits of agreement from the mean VA for the comparisons of Runge vs. ETDRS, Runge vs. Snellen, and Snellen vs. ETDRS charts were ± 0.39, ± 0.57, and ± 0.45 logMAR units. (Values may differ slightly from those calculated from the table due to rounding effect). Values were generally larger for comparisons among subjects with poorer acuity than those with good acuity.

**Table 1:** Bland–Altman Plot values of the estimated 95% limits of agreement for each comparison

Group by visual acuity category	Comparison	Lower limit	Upper limit	95% interval from mean difference
All	Runge vs ETDRS	−0.33 (−0.39; −0.27)	0.46 (0.40; 0.52)	± 0.39
All	Runge vs Snellen	−0.57 (−0.66; −0.49)	0.56 (0.48; 0.64)	± 0.57
All	Snellen vs ETDRS	−0.52 (−0.59; −0.45)	0.38 (0.31; 0.45)	± 0.45
Poor VA	Runge vs ETDRS	−0.38 (−0.46; −0.30)	0.42 (0.33; 0.50)	± 0.40
Poor VA	Runge vs Snellen	−0.69 (−0.81; −0.57)	0.46 (0.34; 0.58)	± 0.58
Poor VA	Snellen vs ETDRS	−0.67 (−0.78; −0.56)	0.40 (0.29; 0.52)	± 0.54
Good VA	Runge vs ETDRS	−0.25 (−0.33; −0.17)	0.48 (0.40; 0.56)	± 0.37
Good VA	Runge vs Snellen	−0.35 (−0.45; −0.25)	0.57 (0.47; 0.67)	± 0.46
Good VA	Snellen vs ETDRS	−0.27 (−0.33; −0.21)	0.28 (0.22; 0.33)	± 0.27

The first value in each cell is the estimated limit, corresponding to the bold blue dashed lines in Fig. 2. Values in parentheses represent the 95% confidence interval of the estimated limit itself, corresponding to the light blue dotted lines in Fig. 2. All values are in logMAR units

## Discussion

Our observations suggest that the Runge card, under controlled conditions, could provide an overall estimate of minimal angle of resolution that approximates that of an electronic Snellen test at distance as determined by its agreement level with the ETDRS distance chart. Their respective correlation coefficients with the ETDRS chart were practically identical, although differences in performance at various acuity levels were observed that might be expected based on chart design. Bias within each comparison (mean visual acuity difference among tests) was small relative to overall variation (as measured by standard deviation around the mean difference) such that the impact of the observed bias in the clinical environment might be relatively small. It is important to note that a Snellen chart was used as a comparator in this study solely to provide a reference point for how the Runge card might perform clinically relative to a commonly used form of testing. The relative clinical utility of each of the charts was not evaluated in this study. Nevertheless, based upon the above findings, we predict that the Runge card might be expected to function at least at a level that is being achieved in current practice using various Snellen charts.

We speculate that at least some of the difference in performance between the electronic Snellen chart and the Runge card relative to the ETDRS chart could relate to the number of letters at each acuity level, which is known to affect repeatability of a test [8]. This effect might also exist when comparing two different charts if they also differed in this respect, adding variation to test performance. Although most Snellen charts have fewer letters per line for worse acuity and more for better acuity, the Runge card has the same number of letters (three) for each visual acuity level. This may explain greater agreement between Runge and ETDRS with poor acuity and lesser at good acuity. Also, unlike ETDRS charts with spacing between acuity lines that is a constant proportion of letter size, spacing between lines increases proportionally with the Runge card as letters become smaller (see Fig. 1), which could lead to less crowding between letters with Runge testing for better acuity than ETDRS charts and contribute to worse agreement between the two tests at good acuity. Variation in testing distance with near (Runge) testing, such as might occur with an inadvertent drifting of a subject closer to the card to resolve smaller letters (decreasing the testing distance), would have a greater effect on angular letter size than a similar slight subject drift when viewing a distance chart. This problem can be resolved by measuring the near testing distance in meters divided by the letter size in M units (on back of Runge card as seen in Fig. 1b) to obtain decimal visual acuity, which can be converted to logMAR or Snellen-equivalent visual acuity. The ETDRS chart has been shown to have relatively low test–retest variability [6, 8–12], and is likely more valid than a Snellen chart for assessing the Runge card. In contrast, Snellen charts differ by manufacturer in letters per line and progression through letter size [13], and have a relatively high test–retest variability [11, 12].

In the current study, for both the Runge and Snellen charts there existed large variability among subjects in how well their results agreed with ETDRS measurements. This variability far exceeded test–retest variability (TRV) reported for the ETDRS chart itself, reported to range from ± 0.07 to ± 0.19 logMAR units [14]. This compares to ± 0.39 logMAR units for the overall comparison between the Runge card and ETDRS chart. Although a larger absolute value would be expected because of differences in methodology from a TRV study, the difference in variability is notable. Kaiser [5] reported increased variability simply by changing the testing distance for the ETDRS chart from four meters and two meters, and speculated that subject fatigue may have played a factor, presumably because most other optical and environmental factors had been highly controlled. He also found that the worse the visual acuity, the greater this effect; we observed the same. Our testing protocol used three different acuity charts and likely required more refractive adjustments; with this more extensive protocol, the effect of fatigue might be further magnified. Chart design and other aforementioned technical issues (e.g., differences in letter progression, numbers of letters per line, variable crowding, varying sensitivity of angular letter size to inadvertent subject drift) would increase variability compared to findings derived from a test-retest variability assessment. Additionally, differences in patient population in terms of general cognition, ocular condition, and degrees of visual loss might also have led to greater variability. The relative contribution, if any, of the above factors to overall variability could not be determined in this study.

This study has several strengths. To eliminate examiner-based variation, a single examiner performed all testing. A protocol refraction correcting for the different working distances was performed to eliminate as much as possible refractive error from assessing the test characteristics of the Runge card. Participants had a range of visual acuities, including some without ocular disease. A nearly equal number of participants had good and poor vision, with pre-defined parameters, allowing meaningful subgroup analysis.

Limitations of our study exist. Correlation between visual acuity tests might be influenced by the type of ocular disease present; a large majority of our patients had retinal disease. Whereas we performed refraction for study purposes, such is not done in primary or urgent care settings; thus, agreement among tests in those settings may differ from our results. The absence of test–retest variability data associated with the Runge card currently inhibits its use as an instrument for clinical outcome trials. As there are fewer letters for each visual acuity level with the Runge card (three) compared with the ETDRS chart (five), results from Runge testing would be expected to be more variable than results from ETDRS testing. In addition, the availability at present of only one Sloan letter version of the Runge card—pediatric adaptations do exist—precludes implementation in longitudinal studies or studies requiring binocular acuity measurement.

Any shortcomings of the Runge card may be outweighed by its ease of use, particularly in a primary or urgent care setting. A reading of just a single line on the card, although not ideal, can give a quick assessment of acuity, an advantage for testing patients with altered consciousness, cognitive impairment, ocular pain, photophobia, or other conditions that can limit the scope and duration of acuity testing. Its portability and easy storage in examination rooms may also enhance its accessibility relative to standard distance testing, particularly for non-ambulatory patients. Our clinical experience with a preliminary implementation of the Runge card with accessory pinhole testing in our emergency department (ED) has resulted in its rapid adoption, with rates of agreement (within two lines of visual acuity) between ED non-ophthalmic technicians and ophthalmology residents being observed in about two-thirds of patients. We consider this encouraging given that the test was administered by up to 100 different ED staff members and nine different residents in a challenging patient population with many clinical variables at play, including test termination and acuity assignment rules that had not yet been standardized between the two groups of examiners. The two-line standard is also stringent, given that the test-retest variation of standardized visual acuity using the ETDRS chart itself is ~ 1–2 lines [12, 14]. We expect further improvement as we refine our ED testing protocol based on our initial findings. Better outcomes and resource utilization may result, given that poor acuity is a strong predictor of severe eye injury and need for specialized ophthalmic evaluation [15].

### Summary

#### What was known before

- Current near visual acuity charts can be difficult to administer in certain primary care settings and possess drawbacks that include nonstandard progression of letter size and chart lines containing letters of varying number and difficulty level.

#### What this study adds

- This study provides evidence of potential utility of the Runge near card by showing clinically acceptable agreement with the standard ETDRS chart that is equivalent to that of the commonly used Snellen chart. Its potential effectiveness may be further supported by its user-friendly characteristics and ease of use.
